# Intraparenchymal Hemorrhage: A Fatal Presentation of Undiagnosed Acute Myelogenous Leukemia

**DOI:** 10.7759/cureus.25592

**Published:** 2022-06-02

**Authors:** Rachelle Hamadi, Marc Assaad, Juda Zurndorfer, Khalil El Gharib, Raymond Kwok, Meekoo Dhar, Alfred Schwab

**Affiliations:** 1 Internal Medicine, Staten Island University Hospital, New York City, USA; 2 Neurology, Staten Island University Hospital, New York City, USA; 3 Oncology, Staten Island University Hospital, New York, USA; 4 Hematology and Oncology, Staten Island University Hospital, New York City, USA

**Keywords:** leukapheresis, intracerebral hemorrhage, hyperleukocytosis, blast crisis, acute myelogenous leukemia

## Abstract

We present the case of a 73-year-old patient who was admitted to the neurocritical care unit with spontaneous intracerebral hemorrhage (ICH). Upon further investigation, she was found to have hyperleukocytosis and thrombocytopenia due to acute myelogenous leukemia (AML), likely resulting in coagulopathy, vessel friability, and consequential intraparenchymal bleed. Prior reports of AML presenting with ICH are scant in the literature. As such, a heightened awareness of such a phenomenon is recommended for rapid detection and appropriate tailored management. This hopefully would, in turn, optimize outcomes.

## Introduction

Spontaneous intracerebral hemorrhage (ICH) is a common cause of stroke, accounting for 10% to 20% of all stroke types, and is often a result of uncontrolled hypertension. The hemorrhage often originates in the smaller perforator arteries, most notably in the basal ganglia, thalamus, pons, and cerebellum. Penetration of the blood compresses surrounding vessels and parenchyma, leading to profound edema, tissue injury, and significant patient disability and mortality [1].

Acute myelogenous leukemia (AML) is the most common type of acute leukemia, and its incidence increases with age [2,3]. The spectrum of clinical presentations is wide ranging. AML patients may be minimally symptomatic but certainly may manifest in extremis with disseminated intravascular coagulopathy (DIC), severe sepsis due to dysfunctional white blood cells (WBC), or significant bleeding due to marked thrombocytopenia. Among all hematological malignancies, ICH is most commonly seen in AML patients [4,5] and harbors a high rate of morbidity and mortality [6]. We herein report a case of a spontaneous ICH in a patient presenting with previously undiagnosed AML in blast crisis.

## Case presentation

This is a case of a 73-year-old female with a past medical history of essential hypertension, dyslipidemia, and right-sided breast cancer, in remission, status post chemoradiotherapy. The patient was sent from a nursing facility at night to the emergency department (ED) with concern for stroke, with an acute change in mental status, lethargy, dysarthria, inability to follow commands, and right upper extremity weakness. The patient’s initial blood pressure (BP) was 150/85 mm Hg and heart rate was 98 beats per minute. She was tachypneic and her oxygen saturation was 96%, with a Glasgow Coma Scale (GCS) of 10 and the National Institutes of Health Stroke Scale (NIHSS) score of 31. Given the patient’s inability to tolerate her secretions, the decision was made to intubate her.

**Figure 1:**
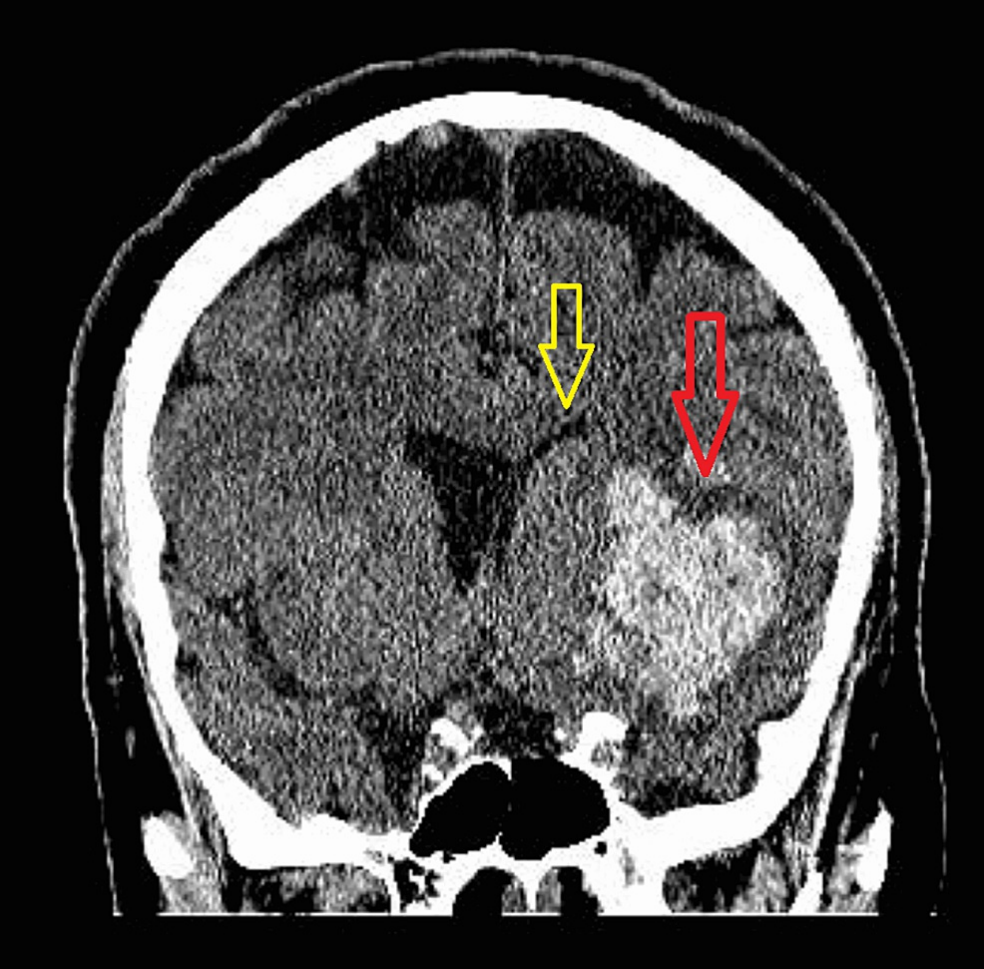
Computed tomography of the head: coronal view showing the effacement of the left lateral ventricle frontal horn and 5-mm left to right midline shift (yellow arrow) secondary to intraparenchymal hemorrhage (red arrow)

Initial computed tomography of the head (CTH) showed a large left anterior temporal lobe and basal ganglia intraparenchymal hematoma (IPH) approximately 27 cubic centimeters with mass effect, and a 5-mm left to right midline shift (Figure 1). CT angiography of the head and neck revealed no vascular anomaly. Neurocritical care was consulted, and the patient was admitted for ICH and respiratory failure. Standard neurocritical care management for ICH was initiated, including BP management, hypertonic fluid for perihematoma edema with shift, seizure prophylaxis, and neurosurgical consultation for the evaluation of possible external ventricular drain placement, which was deferred.

**Figure 2:**
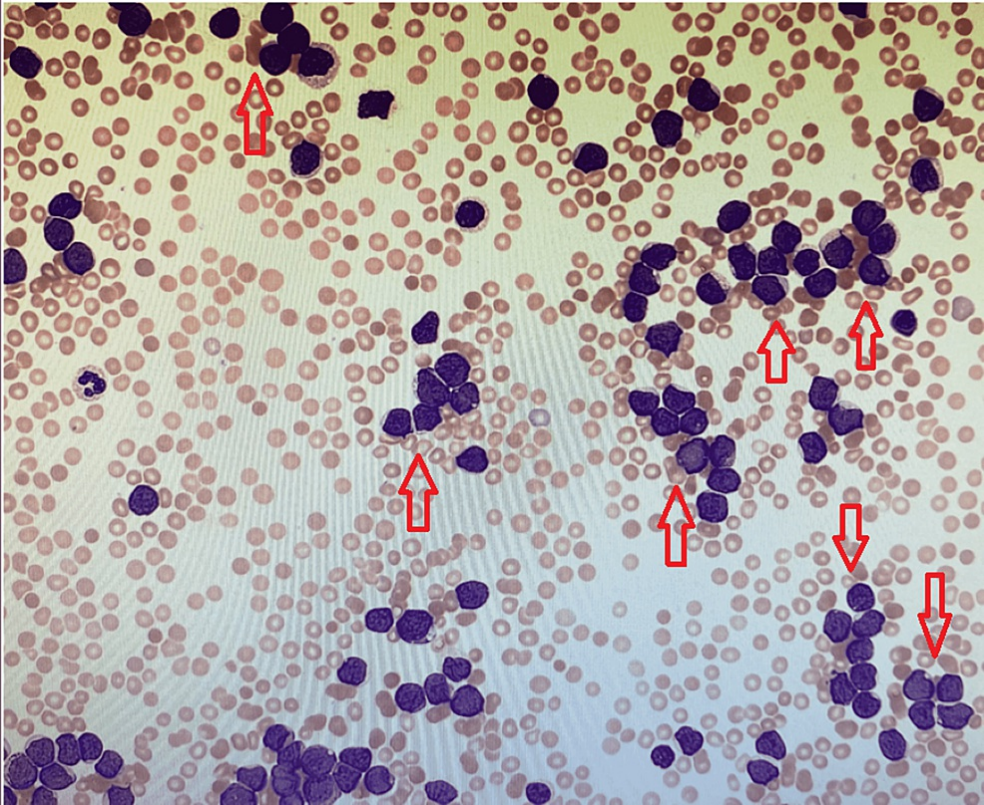
Peripheral smear showing a large number of blasts (arrows).

Initial lab workup was significant for hyponatremia of 128 mmol/L, hypokalemia of 1.8 mmol/L, and an elevated serum lactate dehydrogenase (LDH) of 1204 u/L. Complete blood cell count (CBC) revealed microcytic anemia with a hemoglobin level of 8.9 g/dL, thrombocytopenia with a platelet count of 25 000 per Ul, and, interestingly, a WBC count of 293,000/uL with lymphocytic predominance; DIC panel was negative. Upon review of the outpatient records six months prior, the patient had CBC values that were within normal reference. The patient was transfused platelets; hematology was urgently consulted for leukapheresis and cytoreductive therapy given the concern for acute leukemia and hyperleukocytosis and leukostasis precipitating bleeding. A manual smear differential was ordered, which showed a large number of blasts (Figure 2). The patient was started on allopurinol and intravenous fluids.

**Figure 3:**
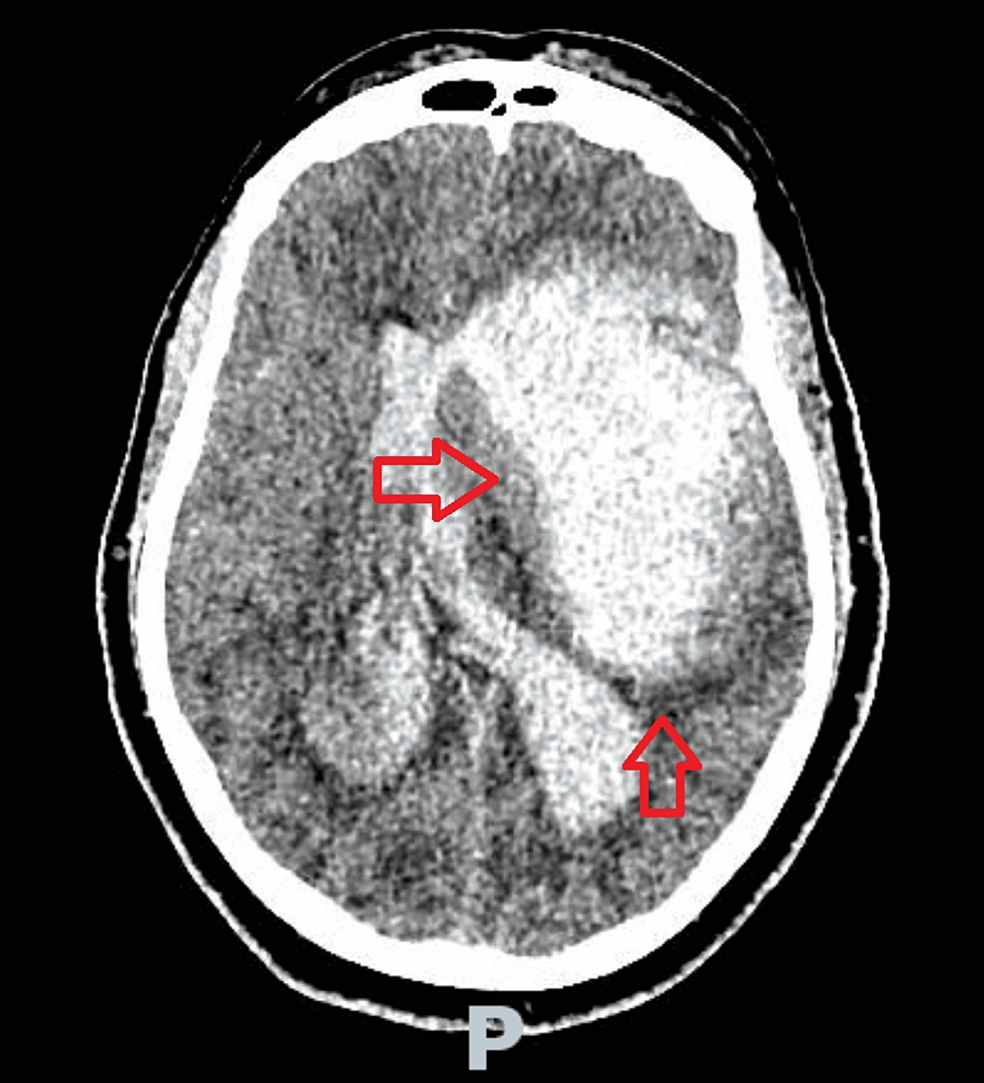
Computed tomography of the head showing large intraparenchymal hemorrhage of the left anterior temporal lobe and left inferior frontal lobe (arrows) with surrounding edema and mass effect.

The patient was brought to the neurocritical care unit. Within a few hours of arriving at the unit, the patient’s neurologic condition began to worsen with further loss of brainstem reflexes. The patient was taken for a repeat CTH (Figure 3). The IPH significantly increased, now with extensive amount of intraventricular hemorrhage, cerebral edema, and uncal and cerebellar herniation. The patient became completely unresponsive and anisocoric, and thus emergency intracranial pressure crisis management was employed including, but not limited to, head of bed elevation and midline placement of the head, hyperventilation, 23.4% hypertonic saline, intravenous mannitol, and neurosurgery consultation for intervention. The patient was determined not to be a good candidate for any surgical intervention given poor prognosis. Despite aggressive acute medical neurocritical care management, the patient proceeded to lose all brainstem reflexes. Given the patient’s poor prognosis and after conferring with family, any further aggressive medical measures such as leukapheresis were not pursued. The patient’s code status was changed to do not resuscitate, and per the family’s wish, the patient was compassionately extubated and soon thereafter expired. On the peripheral smear, the patient was found to have 89.8% blasts (Figure 2). Blood flow cytometry was obtained, which revealed 74% of myeloblasts, which strongly expressed myeloperoxidase (MPO), CD117, CD33, CD13, and partially CD11b, findings compatible with AML. It was several days later that genetic and molecular studies demonstrated translocation of chromosomes 15 and 17, findings consistent with acute promyelocytic leukemia (APL) or AML type 3.

## Discussion

Our patient had a fatal presentation of spontaneous ICH as the presenting manifestation of AML with blast crisis. Spontaneous ICH is a cause of long-term disability or death often because of risk factors including hypertension, smoking, excessive alcohol intake, hyperlipidemia, antithrombotic agents, and illicit drug use, and accounts for 10-20% of all strokes [7].

ICH is a very rare initial presentation of AML; however, it is the second associated leading cause of death in patients with AML [8,9]. The patient had several risk factors contributing to ICH as listed in other studies, which are hypertension, hyperleukocytosis, and thrombocytopenia. Other risk factors are DIC, sepsis, and vessel wall abnormalities [5,8,10]. The mortality rate in such cases exceeds 50%, and death occurs commonly in the first 48 hours [8,11]. Also, in most of the aforementioned cases, ICH develops in the cerebral cortex according to Chen et al. [5,8], in which APL is usually the culprit [5,8,12]. The pathogenesis of ICH in hyperleukocytosis is multifactorial: mechanical obstruction of small vessels, which means more endothelial adhesion and thus more invasion [13]. Additionally, hyperleukocytosis causes hyperviscosity and leukostasis, which lead to hypoxic vasodilation, increased vascular permeability, and rupture of small cerebral vessels [14,15]. Yu et al. in a systematic review found that a baseline leukocyte increase has a significant correlation with poor short- and long-term functional outcome in patients with ICH, as well as a higher mortality rate [16].

Immediate management of acute ICH involves stabilization of airway and hemodynamics including prompt correction of hypertension, rapid neuroimaging, correction of coagulopathies, and neurosurgical intervention [17]. While our patient did not undergo neurosurgical intervention, platelets were still transfused in the setting of thrombocytopenia. The PATCH study found that platelet transfusions were associated with significant increase in adverse events without any decrease in hematoma expansion; however, recent guidelines suggest platelet transfusions for patients undergoing neurosurgical procedures [17]. In our case, the patient was actively bleeding with a platelet count of <50,000 ad thus transfusion was appropriate. Treatment options for hyperleukocytosis include hydroxyurea if the patient is asymptomatic, and chemotherapy or leukapheresis [11] when the patient has symptomatic hyperleukocytosis. Neurosurgical evacuation of hematoma in spontaneous ICH, while debatable, may be beneficial in limited cases according to the STITCH I and STITCH II trials, while leukapheresis efficacy to rapidly filter blasts remains controversial [18,19]. In our patient, the risks involved in emergent craniotomy or craniectomy outweighed the benefits given the high likelihood of intraoperative bleeding. Van De Louw conducted a retrospective study on 90 patients who underwent leukapheresis after the development of ICH and showed no survival benefit [13]. The same findings were reported in a meta-analysis conducted by Oberoi et al [20]. Van De Louw explored the paradoxical worsening effect that leukapheresis may have on pre-existing ICH by promoting DIC from cellular destruction and release of intracellular content [13].

## Conclusions

Despite the mentioned reports of ICH in AML, this phenomenon remains rare and the prognosis is dismal. Our case explores this uncommon entity, highlighting the importance of prompt recognition and urgent implementation of the correct therapeutic modalities, as time prevails over all other prognostic factors.
